# Suppression of Clothing-Induced Acoustic Attenuation in Robotic Auscultation

**DOI:** 10.3390/s23042260

**Published:** 2023-02-17

**Authors:** Ryosuke Tsumura, Akihiro Umezawa, Yuko Morishima, Hiroyasu Iwata, Kiyoshi Yoshinaka

**Affiliations:** 1 Health and Medical Research Institute, National Institute of Advanced Industrial Science and Technology, Tsukuba 305-8564, Japan; 2 Department of Creative Science and Engineering, Waseda University, Tokyo 162-0042, Japan; 3 Faculty of Medicine, University of Tsukuba, Tsukuba 305-8577, Japan

**Keywords:** auscultation, acoustic attenuation, medical robot, gender bias

## Abstract

For patients who are often embarrassed and uncomfortable when exposing their breasts and having them touched by physicians of different genders during auscultation, we are developing a robotic system that performs auscultation over clothing. As the technical issue, the sound obtained through the clothing is often attenuated. This study aims to investigate clothing-induced acoustic attenuation and develop a suppression method for it. Because the attenuation is due to the loss of energy as sound propagates through a medium with viscosity, we hypothesized that the attenuation is improved by compressing clothing and shortening the sound propagation distance. Then, the amplitude spectrum of the heart sound was obtained over clothes of different thicknesses and materials in a phantom study and human trial at varying contact forces with a developed passive-actuated end-effector. Our results demonstrate the feasibility of the attenuation suppression method by applying an optimum contact force, which varied according to the clothing condition. In the phantom experiments, the attenuation rate was improved maximumly by 48% when applying the optimal contact force (1 N). In human trials, the attenuation rate was under the acceptable attenuation (40%) when applying the optimal contact force in all combinations in each subject. The proposed method promises the potential of robotic auscultation toward eliminating gender bias.

## 1. Introduction

Since the 1800s, auscultation has been a crucial aspect of clinical examination. It is a cost-effective screening method for detecting abnormal signs [1] and remains an important tool in the 2020s, as cardiopulmonary disease is a major cause of death and illness, affecting quality of life and healthcare costs [2]. Additionally, recent studies have suggested that auscultation may be used as a diagnostic tool for COVID-19 patients and as a follow-up tool for noncritical COVID-19 patients [3,4].

During auscultation, patients must expose their chests fully, as doctors need to place the stethoscope directly on the skin for accurate diagnostic results. However, this can cause embarrassment and discomfort for patients when exposed to doctors of the different gender and being physically touched, which may also affect the diagnostic quality [5]. In addition, regardless of the gender, taking off their clothes is time-consuming, especially when wearing layers. Moreover, patients often feel cold when taking off their clothes during the examination. Therefore, it is ideal to perform the auscultation over the clothing without physical touching by physicians. We believe that robot technology has the potential to solve these issues because a robot does not have a specific gender and can perform the auscultation remotely. The use of robot-assisted exams, particularly in ultrasonography, has been studied worldwide [6,7]. These systems can be classified into two categories: remote operation and autonomous operation [7,8]. These robot-assisted systems allow for exams to be performed remotely, eliminating the need for physical contact between doctors and patients and avoiding direct contact with the patient’s body. If the robot-assisted system is able to perform auscultation autonomously, it can potentially solve the aforementioned issues caused by taking off clothing. Thus, there is a need for further research and development in robot-assisted auscultation over clothing.

### 1.1. Related Work on Robotic Auscultation

Although many robotic systems for assisting diagnoses, such as ultrasonography, have been developed, there are few studies focusing on auscultation. Falleni et al. developed a tele-operated robotic interface for remote auscultation [9]. The robot places a stethoscope on the patient via remote operation by a physician with a haptic interface, and an RGB-D sensor streams a video of the scene. Yang et al. proposed a multimodal tele-operative robot, which includes the auscultation for COVID-19 prevention and control [10]. Krumpholz et al. developed a tele-operative robot with a unique end-effector which integrates a palpation mechanism and an auscultation device [11]. Those three studies aimed to perform auscultation with their robot systems in telemedicine. On the other hand, there have been two studies focusing on the automation of auscultation with robots. Zhu et al. developed a robotic platform that can obtain clear heart and lung sounds autonomously [12]. The system estimates the initial auscultation location based on the non-rigid registration and adjusts the location with informative path planning using auditory feedback. Lopes et al. developed a collaborative robotic platform targeting auscultation on the back, which enables the estimation of the auscultation area based on the extraction of back features with an RGB-D camera and a deep-learning-based prediction model [13]. MobileNetV2-UNet is used as the prediction model for extracting reference points on the back via the RGB-D camera, and the robot arm reaches the estimated location automatically. However, this study did not obtain sounds with a stethoscope. These two studies also showed its feasibility in human trials.

As the limitation of those studies, auscultation was performed on subjects without their clothing. In order to completely address the issue of gender bias, auscultation should be performed without the need for patients to remove their clothes in terms of their privacy. Additionally, if auscultation can be performed over clothing with a robot, it would provide the benefit of reducing the examination time because it does not require time to change clothes. Casual observation reveals that doctors often auscultate the heart and lungs of the patient through clothing such as a hospital gown. Although the quality of sounds may be decreased by the presence of clothes, this can potentially be improved by adjusting the contact pressure of the stethoscope [14]. Additionally, recent electronic stethoscopes have been shown to be promising options for satisfying diagnostic sound quality, even over clothes [15,16].

### 1.2. Contribution

To eliminate gender bias during auscultation exams, we aimed to develop a robotic platform that enables performing automated auscultation over clothes. Our previous works have developed a prototype of the robotic auscultation platform and have demonstrated the feasibility of auscultation over clothing in a phantom study [17,18]. Moreover, they showed that the power spectrum of the obtained sound is decreased due to clothing and can be improved by adjusting the contact force between the stethoscope and body surface. As the limitation, the results were very preliminary because the experimental condition of clothing used in the previous study was limited. We performed a phantom experiment with only three clothing combinations and did not examine it with quantitative and comprehensive experimental parameters. In order to perform auscultation over clothing with a robotic platform, it is necessary to further investigate the relationship between the applied contact force and clothing condition. In addition, although the prototype was validated in the phantom study, human trials were not performed.

The contribution of this paper is that it addresses those shortcomings and consists of the following three points. First, we analyzed the relationship between acoustic attenuation and clothing condition when performing auscultation over clothing, and we developed a robot system with an end-effector adjusting the contact force safely. Second, we conducted phantom experiments with various types of clothing, which included thickness, material and layering combination. Finally, we performed human trials in several clothing combinations to validate the feasibility of auscultation over clothing.

## 2. Materials and Methods

### 2.1. Acoustic Attenuation due to Clothing

**Figure 1:**
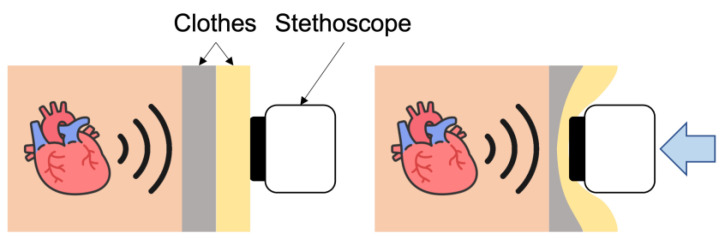
Suppression of clothing-induced acoustic attenuation.

The power level of the obtained sound is known to be decreased due to clothing experimentally. This phenomenon may be caused by acoustic attenuation when transmitting the sound through clothing, as shown in Figure 1. Acoustic attenuation measures the loss of energy as sound propagates through a medium with viscosity. When sound propagates through such a medium, there is always thermal consumption of energy due to viscosity. The acoustic attenuation coefficients of a wide range of viscoelastic materials, including soft tissues, polymers, soils and porous rocks, can be expressed as a power law with respect to frequency, as follows [19,20]:

(1) $$P\left(x+\Delta x\right)=P\left(x\right)e^{-\alpha \left(\omega \right)\Delta x},$$

where ω is the angular frequency, *P* is the acoustic pressure, Δx is the wave propagation distance, and α(·) is the attenuation coefficient. The attenuation coefficient α is related to the medium type, being lower for liquids, such as water, and higher for solids, such as bone [21]. Fabric material also has the characteristic of acoustic attenuation [22,23]. Then, the type of clothing in auscultation affects the degree of acoustic attenuation. The wave propagation distance Δx corresponds to the thickness of clothing. Thus, the acoustic attenuation may be increased exponentially depending on the type and thickness of clothing.

### 2.2. Suppression of Acoustic Attenuation with Robotic Auscultation Platform

#### 2.2.1. Requirements

In order to suppress the aforementioned acoustic attenuation, it is necessary to decrease the wave propagation distance Δx and to select clothing with a low attenuation coefficient α. Compressing the clothing can decrease the thickness of the clothing, which contributes to decreases in Δx. On the other hand, an excessive contact force may not only cause discomfort or pain for patients but can also suppress the vibration of the membrane of the stethoscope, which decreases the quality of the acquired sound. Therefore, it is necessary to apply an optimal contact force that satisfies the acquisition of diagnostically sufficient sound quality while also ensuring patient safety. Because the related work [14] applied a contact force ranging from 30 g to 555 g for auscultation over a clothing, the required contact force in this study was set to the range of 0 to 5 N. Patient safety may be ensured even if the contact force of up to 5 N is applied to the body, because other medical robot systems that require physical contact with the body, such as ultrasonography, apply a contact force of more than 10 N [24,25]. In addition, contact between the stethoscope and the body surface needs to be maintained well during auscultation, in response to the displacement of the body surface, such as by respiratory motions.

#### 2.2.2. System Design

**Figure 2:**
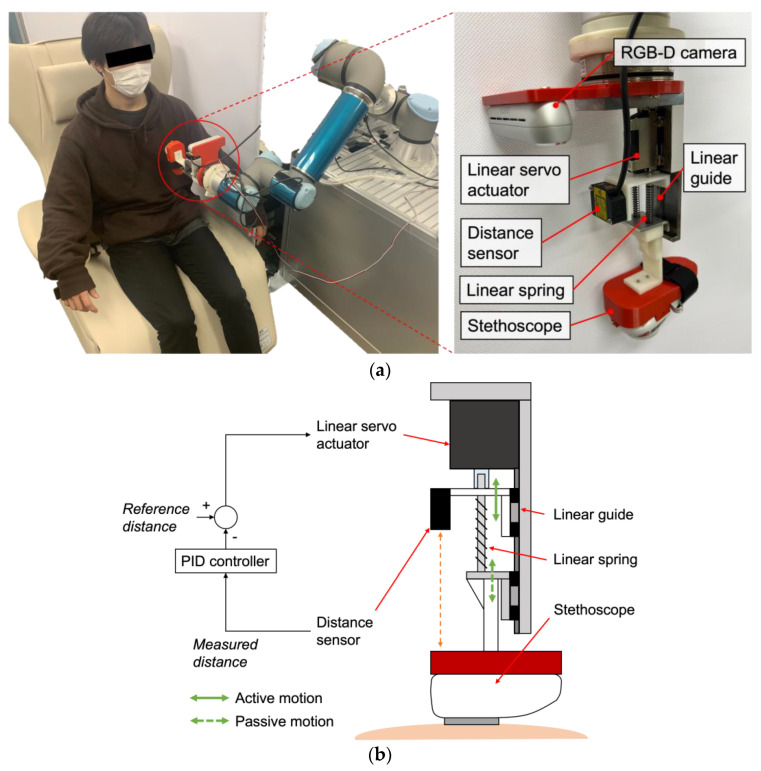
(**a**) Overview of the developed end-effector with a spring-based passive-actuated mechanism; (**b**) System design and control architecture.

To achieve the requirements, a robot system with a unique end-effector with a spring-based passive-actuated mechanism was developed. Figure 2 illustrates the overall design of the developed robot system that is composed of a 6-degree-of-freedom cooperative robot arm UR5e (Universal Robotics, Odense, Denmark) and an end-effector holding a digital stethoscope JPES-01 (MITORIKA, Ibaraki, Japan), which is connected to a host PC via Bluetooth. The role of this end-effector is to maintain an arbitral contact force of the stethoscope against the body surface safely, regardless of the displacement of the body surface, such as by respiratory motions, when placing the stethoscope via the robot arm on the listening positions. The end-effector was designed to adjust the position of stethoscope along the pressing direction in order to maintain the intended contact force while compensating for the displacement. The developed end-effector is composed of a linear servo actuator L12-20PT (MigthyZap, Bucheon, Republic of Korea), a general linear spring, an optical distance sensor ZX-LD100L (Omron, Osaka, Japan) and a linear guide SSE2B6-70 (Misumi, Tokyo, Japan). An upper part connected to the linear servo actuator pushes to a lower part connected to the stethoscope via the springs. The position of the upper part is actively controlled, whereas the lower part is passively moved in response to the displacement of the body surface or the compression of springs by the upper part, as shown in Figure 2b. Thus, the contact force between the body surface and the stethoscope is determined by the amount of compression of the springs. The generated contact force $F_{C}$ is calculated as follows:

(2) $$F_{C}=k\Delta l_{S},$$

where k and $\Delta l_{S}$ represent the linear spring coefficient and the amount of compression of the linear springs, respectively. Then, once the desired contact force is determined, the required spring compression, i.e., the pressing distance controlled by the linear actuator, can be calculated. The compression amount is measured in real-time using the optical distance sensor. The developed end-effector features a linear servo actuator and an optical distance sensor with resolutions of 6.6 μm and 16 μm, respectively, and maximum ranges of 27 mm and 100 mm. Two linear springs are used with a coefficient of 0.45 N/mm. The linear servo actuator is controlled by an Arduino-based controller IR-STS01 (MigthyZap, Bucheon, Republic of Korea) and the optical distance sensor’s value is acquired using a DAQ tool Analog Discovery 2 (Digilent, WA, USA). A custom-designed software system, programmed in Python and running on Visual Studio Code, synchronizes the control of the linear servo actuator with the reading of data from the optical distance sensor. The position of the linear servo actuator is controlled using a proportional–integral–derivative (PID) control scheme, based on feedback from the optical distance sensor. The generated contact force is reproducible and was validated in previous work [18].

### 2.3. Metrics for Acoustic Attenuation

In this study, we quantitatively evaluated the effect of adjusting the contact force to improve the acoustic attenuation caused by clothing. The degree of improvement in acoustic attenuation is defined as the ratio of the amplitude spectrum when auscultated in the naked and clothed state. The amplitude spectrum in each frequency |X[f]| is calculated by applying a fast Fourier transform to the acquired sound data. In this study, as the frequency used for the auscultation ranges from 0 to 200 Hz, the sum of the amplitude spectrum in the range is used for the calculation of the acoustic attenuation ratio, as follows:

(3) $$Attenuation\;Ratio=1-\frac{\int_{0}^{200}\left|X_{clothed}\left[f\right]\right|df}{\int_{0}^{200}\left|X_{naked}\left[f\right]\right|df}$$

The procedure for sound acquisition is as follows: (i) a certain contact force is applied to the body; (ii) sound pressure data are acquired for 5 s and are digitized at 44,100 Hz by using an electrical stethoscope; (iii) the amplitude spectrum in each frequency is calculated with the acquired pressure data, and a moving average filter is applied to the calculated amplitude spectrum; and (iv) the applied contact force is changed in a certain range, and (ii)–(iii) are repeated.

### 2.4. Experimental Protocol

In this study, we performed four experiments. First, in order to determine an acceptable acoustic attenuation in terms of diagnosis, a subjective evaluation with physicians was conducted. We asked four physicians to score 20 samples of data of abnormal cardiac and respiratory sounds, which were generated by artificially attenuating the source audio file. The acoustic attenuation ratio used in this experiment ranged from 0% to 90%. The score was between 0 and 10. A score of 0 shows that the sound cannot be used for diagnosis at all, and 10 shows that the sound can be heard clearly and can be used for diagnosis.

Next, we investigated the effect of clothing thickness on the acoustic attenuation. In this experiment, shirts of two different materials (cotton and polyester) were prepared with four different thicknesses (1, 5, 10 and 20 mm). A commercial auscultation simulator (Choushin-kun, Sakamoto model, Japan) was utilized to generate cardiac sounds in this experiment. To the simulator covered with the prepared clothes, six-way contact forces (0, 1, 2, 3, 4 and 5 N) were applied by using the developed end-effector.

In the third experiment, several clothing combinations assumed for daily life were prepared. The prepared clothing included shirts, hoodies, and coats, and the combinations used in this experiment were as follows: (i) only a shirt; (ii) shirt + hoodie; and (iii) shirt + hoodie + coat. Similar to the previous experiment, six-way contact forces were applied by using the developed end-effector.

Finally, human trials were performed with four male volunteers. The prepared clothing included a shirt and hoodie, and the experimental scenario was as follows: (i) no clothing; (ii) only the inner shirt; and (iii) inner shirt + hoodie. A six-way contact force (0, 1, 2, 3, 4 and 5 N) was applied around the body surface on the mitral valve. The study protocol was approved by the Institutional Review Board of National Institute of Advanced Industrial Science and Technology (No. 2022-1231), and informed consent was obtained from each volunteer.

## 3. Results

### 3.1. Acceptable Attenuation Ratio

Table 1 and Table 2 show the results of the scoring of abnormal cardiac and lung sounds by the physicians. In all subjects, the score tended to be decreased depending on the acoustic attenuation rate. On the other hand, confidence in the diagnosis of the sound varied based on the individual subjects. The scores of subjects A and B dropped suddenly after 80% and 90%, and the scores of subjects C and D gradually decreased. In both experiments, the scores began to decline after around 40%. Then, we determined the attenuation rate of 40% as the threshold for acceptable attenuation in this study.

### 3.2. Effect of Thickness of Clothing

**Figure 3:**
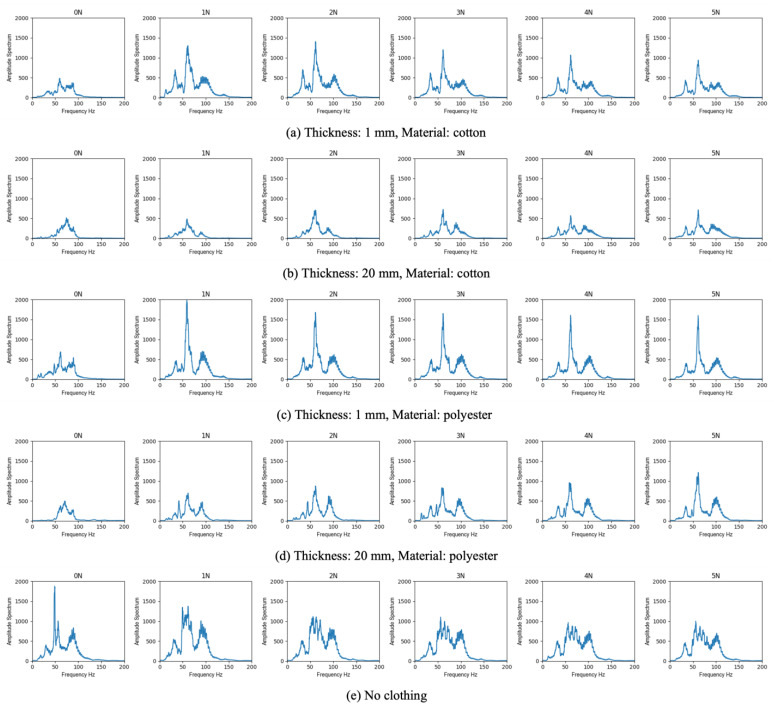
Representative results of amplitude spectrum at each frequency with various thicknesses.

Figure 3 represents the representative results of the amplitude spectrum at each frequency from the experiments with the cotton and polyester shirts with varied thicknesses. Moreover, Table 3 shows the sum of the amplitude spectrum and the attenuation ratio in each condition. We used the maximum amplitude spectrum in the no clothing condition as the basis for calculating the attenuation ratio in this experiment. The results indicate that the sum of the amplitude spectrum decreased as the thickness increased in both shirts. Comparing the results varying in thickness between 1 mm and 20 mm for cotton and polyester shirts under the application of the contact force of 1 N, the attenuation rate increased 55% and 39%, respectively. Moreover, there was an optimum contact force in each of the thickness conditions. Focusing on the results of 1 mm thick cotton and polyester shirts, the attenuation rate decreased 48% and 38% when applying the optimal contact force (1 N) compared to the insufficient force (0 N). When the contact force exceeded the optimum force, the sum of the amplitude spectrum began to decrease. The optimum force tended to increase depending on the thickness. In addition, when comparing the cotton and polyester shirts, the sum of the spectrum of the cotton shirt was lower than that of the polyester shirt.

### 3.3. Validation of Clothing for Daily Life

**Figure 4:**
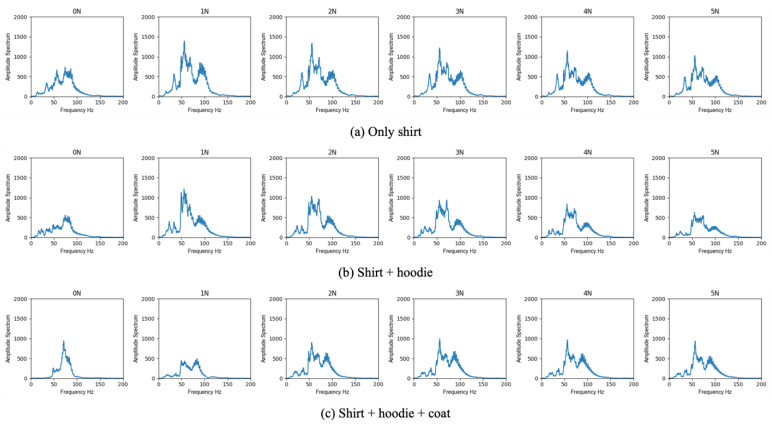
Results of amplitude spectrum at each frequency in the experiment with daily life clothing.

Figure 4 represents the results of the amplitude spectrum at each frequency from the experiments with daily life clothing, combining a shirt, hoodie and coat. Moreover, Table 3 shows the sum of the amplitude spectrum and the attenuation ratio in each condition. Bold fonts in Table 3 represent results above the acceptable attenuation rate of 40% introduced in the first experiment. The results show that the attenuation ratio increased as the number of layers of clothes increased. Similar to the results of the previous experiment, there was an optimum contact force in each of the combination patterns, and the optimum force increased as the number of layers of clothes increased. Comparing the conditions of a shirt; layering a shirt and hoodie; and layering a shirt, hoodie and coat, the optimal contact force and attenuation rate were 1 N and 8%, 1 N and 23%, and 3 N and 38%, respectively. In terms of the acceptable attenuation ratio, there was the case of hearing an acceptable sound with an attenuation rate under 40%, even under the condition of the shirt, hoodie and coat combination by applying the optimum contact force (3 N).

### 3.4. Validation in Human Trials

**Figure 5:**
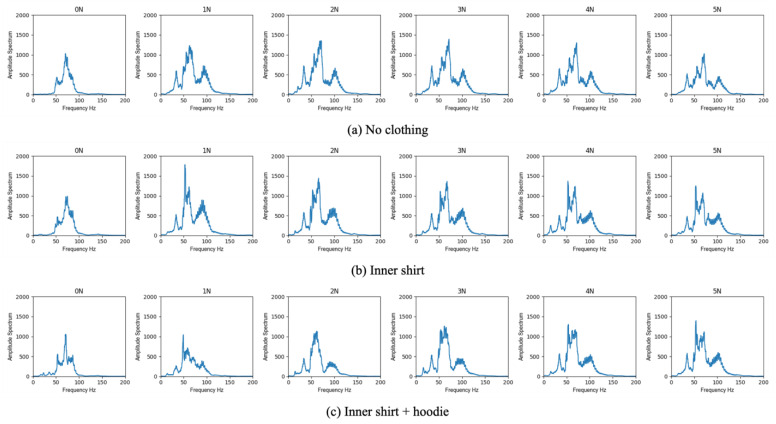
Representative result (subject B) of the amplitude spectrum at each frequency.

Figure 5 shows the representative results of the amplitude spectrum at each frequency with daily life clothing, combining an inner shirt and hoodie in human trials. Moreover, Table 4 shows the sum of the amplitude spectrum and the attenuation ratio in each condition. Bold fonts in Table 4 represent the peak values under applying the optimum contact force in the conditions of an inner shirt and the combination of an inner shirt and hoodie. We used the maximum amplitude spectrum in the no-clothing condition as the basis for calculating the attenuation ratio in each subject experiment. The results show that the sum of the amplitude spectrum decreased as the number of layers of clothing increased, and there was an optimum contact force that improved the amplitude spectrum in all the conditions, similar to the results of the phantom study. Moreover, there were individual differences in the amplitude spectrum between subjects. The absolute value of the sum of the amplitude spectrum for subject A was slightly lower than those for the other subjects. The optimum contact force tended to increase when increasing the number of the layers of clothing, but in the result of subject C, the optimum force was slightly decreased in the condition of an inner shirt compared to the no-clothing condition.

In all combinations in each subject, the attenuation rate was under acceptable attenuation (40%) when applying the optimal contact force. Especially, in case of wearing only inner shirt, there was little difference in the attenuation rate compared to the case of the no-clothing condition. The maximum attenuation rate under applying the optimum contact force was 8%, which the physicians recognized with difficulty.

## 4. Discussion

The results of this study demonstrate the feasibility of robotic auscultation over clothing through the validation of a phantom study and human trials. Although the sound obtained over the clothing was attenuated, especially when increasing the number of layers of clothing, the diagnosable sound could be obtained by adjusting the contact force in some cases. This may be because the wave propagation distance is reduced by compressing the clothing materials, as hypothesized in the Materials and Methods section. It is, of course, difficult to obtain a qualified sound in the case of extreme layering, such as a combination of a shirt, hoodie and coat, but it can be acceptable to have auscultation while wearing a light shirt in terms of acoustic attenuation due to clothing. This finding contributes to the gender-related issues mentioned in the introduction. Prior studies have found that gender can impact the performance of an examination. For example, a study found that male medical students performed worse than female peers in cardiac and pulmonary examinations on female patients [26]. Another study found that both male and female doctors, but especially male doctors, were less likely to correctly perform the cardiovascular exam on female patients, particularly during auscultation in the tricuspid and mitral areas, which are close to the breast [27]. Therefore, if a fully automated robot system performing auscultation over clothing is developed, the gender-related issues derived from both patients and physicians can be solved.

Comparing the results of the cotton and polyester shirts, there was a difference in the obtained amplitude spectrum between those materials. This may be due to the difference in the attenuation coefficients of the materials. Because fiber materials with sound-absorbing properties are known from the perspective of soundproofing [22,23], it may be worth considering fiber materials with low sound-absorbing properties as suitable clothing for auscultation. We also assumed that acoustic attenuation occurred homogeneously and also could be suppressed homogeneously by the compression. Moreover, depending on the type of material and the thickness of the clothing, acoustic attenuation may occur in specific frequency bands. Therefore, it is necessary to focus on the change in sound power due to clothing in a specific frequency utilized for a diagnosis, and to apply a bandpass filter for the intended specific frequency. Moreover, a resonance effect due to the material and the thickness of the clothing should be taken into account for the design of the applied filter.

In this study, we used the sum of the amplitude spectrum as the metric of acoustic attenuation due to clothing. This metric simply represents the volume of the obtained sound. We assumed that the volume sound is related to audibility during auscultation, but it may be possible to focus on sounds in specific frequency bands that are directly related to the diagnosis. For example, heart murmurs have spectral components very concentrated in the band around 600 Hz [28]. In addition, although the acceptable attenuation ratio was determined by the preliminary test in this study, the threshold should be considered for more physicians, as the audible range may vary from physician to physician. Moreover, because of utilizing the spectrum amplitude at each frequency and the sum of the spectral amplitudes of the obtained sound data as the metrics in this study, it was difficult to simply compare the results with a statistical analysis. For evaluating the results statistically, we may need to consider another metric for evaluating the sound quality.

Unlike the related papers regarding robotic auscultation [11,12,13,14,15], the novelty of this study is that it develops a gender-free diagnosis platform performing autonomous auscultation over clothing and solves acoustic attenuation due to the clothing by applying an optimal contact force. Compared to our previous work [18], this study demonstrates the solid feasibility of robotic auscultation over clothing through validation with phantom studies with various type of clothing, which included thickness, material and layering combination, and with human trials with several clothing combinations.

There are several limitations in this study. First, the number of subjects in the human trials was very limited. Although we confirmed the feasibility, it is necessary to perform large-scale human trials to show the efficacy of using this system in clinical situations. Particularly, we did not address the case of female subjects specifically in this study. Because female subjects wear bras, it is necessary to know the effects of straps, underwires and other bra components on acoustic attenuation and noise in order to apply this system to subjects of various genders. Moreover, regarding experiments with female subjects, because this study reveals the feasibility of auscultation over clothing without physical contact by physicians using a robot, it would lower the bar for experiments with female subjects. In addition, it may be worth comparing skinny and fatty subjects because the acoustic propagation distance differs between them. In the case of fatty subjects, compression may contribute to decreases in the acoustic propagation distance because the thoracic surface can be largely deformed by the contact force. After validation with healthy subjects, we need to conduct a clinical trial. In addition, we did not implement an autonomous search algorithm to determine the optimum contact force based on the feedback of the obtained sound in the robotic system. Through the findings in this study, we recognize that the optimum contact force is different due to the thickness and material of clothing and individual difference in subjects. In order to develop practical applications dealing with various conditions, we will implement the online contact force adjustment system based on the obtained sound.

## 5. Conclusions

For patients who are often embarrassed and uncomfortable when exposing their breasts and having them touched by physicians of different genders during auscultation, we are developing a robot for auscultation over clothing. This paper investigates clothing-induced acoustic attenuation quantitively and develops a suppression method for it. Acoustic attenuation can be suppressed by applying an optimal contact force and compressing the clothing, which was validated through phantom and human experiments. In the phantom experiments of 1 mm thick cotton and polyester shirts, the attenuation rate was improved maximumly by 48% and 38% when applying the optimal contact force (1 N) compared to the insufficient force. In the human trials, the attenuation rate was also under acceptable attenuation (40%) when applying the optimal contact force in all combinations in each subject. The findings in this study have the potential to address gender-related issues in auscultation.

For the limitations, the number and variety of subjects were limited, and an autonomous search algorithm to determine the optimum contact force based on feedback of the obtained sound in the robotic system was not implemented. In the future, we plan to increase the number of trials, include female subjects, adopt a hardware configuration closer to that of clinical practice and incorporate a control algorithm that searches for optimal sounds.

## Figures and Tables

**Table 1:** Scoring of abnormal cardiac sounds by physicians.

Attenuation Rate	Subject A	Subject B	Subject C	Subject D
0%	10	10	10	10
10%	10	10	10	10
20%	10	10	10	10
30%	10	10	10	9
40%	10	10	9	9
50%	10	10	9	7
60%	10	10	8	7
70%	10	10	7	6
80%	7	7	7	5
90%	6	5	4	3

**Table 2:** Scoring of abnormal lung sounds by physicians.

Attenuation Rate	Subject A	Subject B	Subject C	Subject D
0%	10	10	10	10
10%	10	10	10	10
20%	10	10	10	10
30%	10	10	9	8
40%	9	10	8	8
50%	9	10	8	7
60%	9	10	8	7
70%	9	8	6	6
80%	8	7	4	6
90%	2	5	2	3

**Table 3:** Sum of amplitude spectrum in each condition in the phantom study ^1^.

		Contact Force N					
		0	1	2	3	4	5
No Clothing		5.94 (30%)	8.48 (-)	8.08 (5%)	7.42 (12%)	7.13 (16%)	6.63 (22%)
Cotton Shirt	1 mm	2.43 (71%)	6.55 (23%)	6.38 (25%)	5.42 (36%)	4.76 (44%)	4.22 (50%)
	5 mm	2.14 (75%)	2.86 (66%)	3.69 (56%)	4.00 (53%)	3.97 (53%)	3.85 (55%)
	10 mm	2.22 (74%)	3.01 (65%)	3.56 (58%)	4.23 (50%)	3.79 (55%)	3.56 (58%)
	20 mm	1.97 (77%)	1.87 (78%)	2.77 (67%)	2.95 (65%)	2.86 (66%)	3.30 (61%)
Polyester Shirt	1 mm	3.33 (61%)	6.56 (23%)	6.49 (23%)	6.13 (28%)	5.56 (34%)	5.19 (39%)
	5 mm	1.94 (77%)	5.59 (34%)	6.85 (19%)	6.62 (22%)	5.89 (31%)	5.20 (39%)
	10 mm	1.95 (77%)	4.66 (45%)	5.55 (35%)	5.58 (34%)	5.55 (35%)	5.25 (38%)
	20 mm	1.90 (78%)	3.19 (62%)	3.84 (55%)	4.28 (49%)	4.47 (47%)	4.89 (42%)
Only Shirt		4.72 (44%)	7.84 (8%)	7.38 (13%)	6.93 (18%)	6.40 (24%)	5.95 (30%)
Shirt + Hoodie		3.43 (60%)	**6.56 (23%)**	**6.23 (27%)**	**5.81 (31%)**	4.86 (43%)	3.83 (55%)
Shirt + Hoodie + Coat		2.81 (67%)	2.91 (66%)	5.12 (40%)	**5.26 (38%)**	5.10 (40%)	4.71 (44%)

^1^ Unit of the sum of amplitude spectrum is ×10^6^ a.u., and parentheses show the acoustic attenuation rate. Bold fonts represent results above the acceptable attenuation rate in the experiment with daily life clothing.

**Table 4:** Sum of amplitude spectrum in each condition in the human trials ^1^.

		Contact Force N					
		0	1	2	3	4	5
Subject A	No clothing	4.59 (2%)	4.69 (-)	4.67 (0%)	4.12 (12%)	4.19 (11%)	4.11 (12%)
	Inner	4.08 (13%)	**4.37 (7%)**	4.28 (9%)	4.29 (9%)	3.83 (18%)	3.92 (16%)
	Inner + hoodie	3.44 (27%)	3.41 (27%)	3.58 (24%)	3.54 (25%)	**3.6 (23%)**	3.53 (25%)
Subject B	No clothing	3.44 (54%)	6.98 (7%)	7.51 (-)	7.35 (2%)	6.90 (8%)	5.34 (29%)
	Inner	3.69 (51%)	7.25 (4%)	**7.44 (1%)**	7.17 (5%)	7.06 (6%)	6.25 (17%)
	Inner + hoodie	3.17 (58%)	4.13 (45%)	5.27 (30%)	6.64 (12%)	7.09 (6%)	7.09 (6%)
Subject C	No clothing	3.73 (53%)	7.40 (8%)	7.97 (0%)	8.00 (-)	7.81 (2%)	7.24 (9%)
	Inner	3.77 (53%)	**7.56 (5%)**	7.49 (6%)	7.24 (9%)	6.68 (17%)	6.18 (23%)
	Inner + hoodie	3.69 (54%)	4.65 (42%)	5.13 (36%)	**5.67 (29%)**	5.11 (36%)	4.41 (45%)
Subject D	No clothing	3.95 (51%)	8.13 (-)	7.92 (3%)	7.53 (7%)	6.83 (16%)	6.11 (25%)
	Inner	2.97 (63%)	7.18 (12%)	**7.50 (8%)**	7.09 (13%)	6.45 (21%)	5.90 (27%)
	Inner + hoodie	2.89 (64%)	5.39 (34%)	**5.48 (33%)**	5.03 (38%)	4.75 (42%)	4.22 (48%)

^1^ Unit of the sum of amplitude spectrum is ×10^6^ a.u., and parentheses show the acoustic attenuation rate. Bold fonts represent the peak values under applying the optimum contact force in the conditions of an inner shirt and the combination of an inner shirt and hoodie.

## Data Availability

Not applicable.
